# Antiproliferative Activity of a New Quinazolin-4(3*H*)-One Derivative via Targeting Aurora Kinase A in Non-Small Cell Lung Cancer

**DOI:** 10.3390/ph15060698

**Published:** 2022-06-02

**Authors:** Ji Yun Lee, Huarong Yang, Donghwa Kim, Kay Zin Kyaw, Ruoci Hu, Yanhua Fan, Sang Kook Lee

**Affiliations:** 1 Natural Products Research Institute, College of Pharmacy, Seoul National University, Seoul 08826, Korea; jiyunkr0@naver.com (J.Y.L.); dkim0719@snu.ac.kr (D.K.); dawkayzinkyaw@gmail.com (K.Z.K.); 18502407040@163.com (R.H.); 2 The Key Laboratory of Chemistry for Natural Products of Guizhou Province, Chinese Academy of Sciences, Guiyang 550014, China; 2018087@stu.gzy.edu.cn; 3 State Key Laboratory for Functions and Applications of Medicinal Plants, Guizhou Medical University, Guiyang 550014, China

**Keywords:** non-small cell lung cancer, aurora kinase A, quinazolin-4(3*H*)-one derivative, G_2_/M cell cycle arrest, EGFR-TKI, combination therapy, EGFR-TKI resistance

## Abstract

Non-small cell lung cancer (NSCLC) is the most common lung cancer subtype. Although chemotherapy and targeted therapy are used for the treatment of patients with NSCLC, the survival rate remains very low. Recent findings suggested that aurora kinase A (AKA), a cell cycle regulator, is a potential target for NSCLC therapy. Previously, we reported that a chemical entity of quinazolin-4(3*H*)-one represents a new template for AKA inhibitors, with antiproliferative activity against cancer cells. A quinazolin-4(3*H*)-one derivative was further designed and synthesized in order to improve the pharmacokinetic properties and antiproliferation activity against NSCLC cell lines. The derivative, BIQO-19 (Ethyl 6-(4-oxo-3-(pyrimidin-2-ylmethyl)-3,4-dihydroquinazolin-6-yl)imidazo [1,2-a]pyridine-2-carboxylate), exhibited improved solubility and antiproliferative activity in NSCLC cells, including epidermal growth factor receptor–tyrosine kinase inhibitor (EGFR-TKI)-resistant NSCLC cells. BIQO-19 effectively inhibited the growth of the EGFR-TKI-resistant H1975 NSCLC cells, with the suppression of activated AKA (p-AKA) expression in these cells. The inhibition of AKA by BIQO-19 significantly induced G_2_/M phase arrest and subsequently evoked apoptosis in H1975 cells. In addition, the combination of gefitinib and BIQO-19 exhibited synergistic antiproliferative activity in NSCLC cells. These findings suggest the potential of BIQO-19 as a novel therapeutic agent for restoring the sensitivity of gefitinib in EGFR-TKI-resistant NSCLC cells.

## 1. Introduction

Lung cancer is the leading cause of death from cancer, and constitutes almost 25% of all cancer-related deaths [1]. Lung cancer is typically subdivided into non-small cell lung cancer (NSCLC) and small cell lung cancer (SCLC). NSCLC comprises of 85% of lung cancer cases and is characterized by diverse gene mutations, including epidermal growth factor receptor (EGFR), B-Raf oncogene (B-RAF), and anaplastic lymphoma kinase (ALK) [2,3]. EGFR mutation is one of the most frequently observed mutations in Asian patients with cancer, and is diagnosed in approximately 50% of the patients [4,5]. EGFR mutations activate its intrinsic tyrosine kinase activity, which subsequently enhances intracellular signaling pathways associated with the growth of NSCLC [4]. Therefore, tyrosine kinase inhibitors that target mutated EGFR have been developed for treating NSCLC, as well as other cancers. The first generation of EGFR tyrosine kinase inhibitors (EGFR-TKIs), including gefitinib and erlotinib, were developed for the treatment of patients with EGFR mutation; however, after several months of treatment, secondary EGFR mutations (T790M) occur that confer resistance to EGFR-TKIs [6,7,8]. Recently, the third-generation EGFR-TKI osimertinib was approved for T790M-positive patients to circumvent the acquired resistance phenotype of EGFR-TKIs. Although the clinical response was improved by osimertinib, triple EGFR mutations occurred in patients with NSCLC that resulted in acquired resistance to osimertinib [9,10]. Thus, overcoming acquired TKI resistance remains a major challengeable for NSCLC treatment. As acquired resistance is associated with diverse factors, searching for promising targets to overcome resistance is an active area of investigation. Recent studies have indicated that aurora kinase is highly activated in acquired-resistant NSCLC cells and involved in the acquisition of resistance to EGFR-TKIs [11,12]. Therefore, aurora kinase represents a plausible target for the management of the EGFR-TKIs-resistant NSCLC cells.

Aurora kinase is a serine/threonine kinase which plays an important role in cell cycle regulation [13]. The highest expression and activity of aurora kinase occur during mitosis, in which it regulates cell division, including chromosomal alignment and segregation [14,15]. Three aurora kinase isozymes are present in human cells: aurora kinase A, aurora kinase B, and aurora kinase C [16]. Aurora kinase A controls centrosome maturation and separation, aurora kinase B regulates chromosome alignment and cytokinesis, and aurora kinase C is involved in spermatogenesis, which regulates meiotic spindles [15]. As the functions of aurora kinases are significantly associated with cell cycle regulation and cell growth, aurora kinases are attractive as putative targets for anticancer agents. Aurora kinase A and B are frequently overexpressed in cancer cells compared with normal cells [17,18]. Inhibition of aurora kinases suppresses cell proliferation by arresting the cell cycle in mitosis, and subsequently results in apoptotic cell death [19,20]. Based on this evidence, aurora kinase inhibitors have been developed that exhibit significant antiproliferative activity in various cancers, including lung cancer, breast cancer, chronic myeloid leukemia, and colorectal cancer [21,22,23]. The association of aurora kinases in the proliferation of NSCLC cells has been reported, and inhibition of aurora kinase A has been shown to result in decreased proliferation of EGFR-TKI-resistant NSCLC cells, which possess active aurora kinases [24,25,26]. Subsequent clinical trials have been carried out with aurora kinase A inhibitors in EGFR-TKI-resistant NSCLC. For example, the combination of osimertinib and alisertinib, an aurora kinase A inhibitor, was used in a phase I clinal trail of EGFR-mutant lung cancer, and a randomized phase III clinical trial of alisertinib was performed for relapsed or refractory peripheral T-cell lymphoma [27,28,29]. Although several compounds that target aurora kinase A are under development, no aurora kinase A inhibitors have been approved for patients with cancer due to the narrow therapeutic window and adverse side effects [30]. Therefore, the development of new safe and effective small-molecule aurora kinase A inhibitors for EGFR-TKI-resistant NSCLCs is needed.

Quinazolin-4(3*H*)-one is a well-known core heterocycle pharmacophore used in medicinal chemistry that exhibits a variety of biological activities, including sedative, antiviral, antibacterial, anti-inflammatory, and anticancer effects [31,32,33,34,35,36]. Quinazolin-4(3*H*)-one derivatives such as quinazolin-4(3H)-one linked to 1,2,3-triazoles and imidazolone-fused quinazolinones exhibit antiproliferative activity against various tumor types, including colorectal cancer, hepatocellular carcinoma, breast cancer, stomach cancer, and lung cancer [35,37]. Certain quinazolin-4(3*H*)-one derivatives exert potent growth-inhibitory activity in lung cancer cell lines by binding to the active site of EGFR [38]. The cytotoxicity of quinazolin-4(3*H*)-one based hydroxamic acid derivatives in cancer cells has been reported as well, and resulted in the inhibition of the histone deacetylase and phosphoinositide 3-kinase mediated signaling pathways [35,39,40]. In addition, many aurora kinase inhibitors contain quinazoline ring structures [41]. In our previous study, we developed a series of quinazolin-4(3*H*)-one inhibitors for aurora kinase A that exhibit antiproliferative activity against cancer cells [42]. From these previous findings, we determined that improving the pharmacokinetic properties of these compounds was necessary in order to generate druggable candidates based on quinazolin-4(3*H*)-one derivatives.

In the present study, we designed and synthesized a novel quinazolin-4(3*H*)-one derivative, BIQO-19, with improved solubility and pharmacokinetic properties and which exhibits increased antiproliferative activity against cancer cells with aurora kinase A inhibition. BIQO-19 can be considered an effective antiproliferative compound for EGFR-TKI-resistant NSCLC cells. Additionally, BIQO-19 exhibits synergistic antiproliferative activity in combination with gefitinib.

## 2. Results

### 2.1. Synthesis and Pharmacokinetic Profile of the Quinazolin-4(3H)-One Derivative BIQO-19

**Figure 1:**
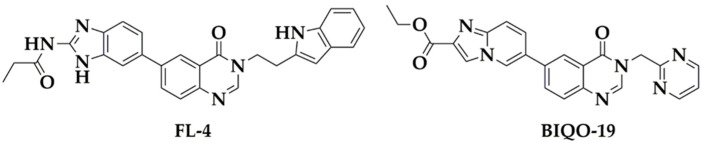
Chemical structures of FL-4 and BIQO-19.

**Scheme 1:**
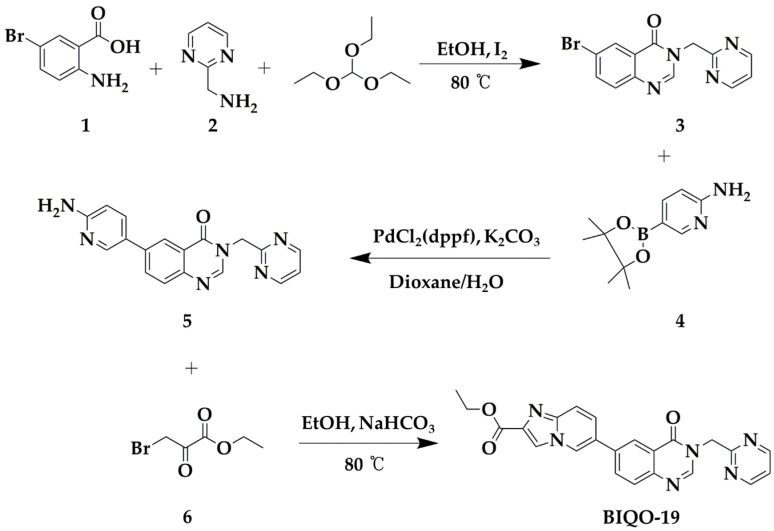
Synthetic method for BIQO-19.

The pharmacokinetic profile of drug candidate compounds is important during the early stages of the drug discovery process. In order to improve the pharmacokinetic profiling of FL-4, which was previously reported as a quinazolin-4(3*H*)-one derivative aurora kinase A inhibitor, we designed and synthesized BIQO-19 (ethyl 6-(4-oxo-3-(pyrimidin-2-ylmethyl)-3,4-dihydroquinazolin-6-yl)imidazo [1,2-a]pyridine-2-carboxylate) as an FL-4 derivative (Figure 1). Based on our previous findings, the tryptamine fragment formed hydrophobic interactions with aromatic amino acids in the active site of aurora kinase A. In order to improve the solubility of this substituent while maintaining its aromatic properties, we replaced the tryptamine moiety with pyrimidine, which is an aromatic ring with higher solubility. Moreover, we introduced imidazo [1,2-a], a pyridine moiety, guided by the skeleton transition principle and using the CADD software SPARK version 10.6.0 (Cresset BioMolecular Discovery Ltd., Welwyn Garden City, UK). Initially, the synthesis of BIQO-19 was derived from a similar synthetic root as the preparation of the previously-reported quinazolin-4(3*H*)-one derivative FL-4 [42] (Scheme 1). The synthetic routes and reagents for the synthesis of compound BIQO-19 are briefly shown in Scheme 1.

To primarily evaluate the pharmacokinetic profiles of FL-4 and BIQO-19, in silico prediction of ADMET properties was performed. As shown in Table 1, BIQO-19 (−4.1 < ADMET_Solubility < −2.0, good solubility) exhibited greater aqueous solubility compared with FL-4 (−8.0 < ADMET_Solubility < −6.0, very low solubility). The model with descriptors AlogP98 and PSA 2D was used for the accurate prediction of the cell permeability of BIQO-19 and FL-4. Based on the model, the criteria for optimum cell permeability for a compound were followed as PSA < 140Å^2^ and AlogP98 < 5. As a result, both BIQO-19 and FL-4 showed good cell permeability. Moreover, BIQO-19 (Level 3, Low penetration) was predicted to penetrate the blood brain barrier more efficiently compared to FL-4 (Level 4, undefined). ADMET CYP2D6 binding predicts whether a compound is a cytochrome P450 2D6 enzyme inhibitor; neither BIQO-19 nor FL-4 was predicted as a P450 2D6 enzyme inhibitor. Potential human hepatotoxicity was predicted using the ADMET hepatotoxicity model. There is considered to be no hepatotoxicity when the value is below −4.154. As shown in Table 1, the value of BIQO-19 was closer to −4.154 than FL-4, indicating less hepatotoxicity. As only the free drug exhibits pharmacological effects, the ADMET plasma protein binding property was calculated using the ADMET_EXT_PPB model. If the value is below −2.209, the compound is predicted to be a weak or non-binder (<90%). Otherwise, it is predicted to be a binder (≥90%); higher values indicate a stronger bond. The plasma protein binding of BIQO-19 (ADMET-EXT-PPB level −0.242) showed weaker binding to the plasma protein than of FL-4 (ADMET-EXT-PPB level is 3.549), suggesting that BIQO-19 has better bioavailability (Table 1). These data indicate that BIQO-19 possesses superior ADMET parameters and druggability compared with FL-4 based on their respective pharmacokinetic profiles.

### 2.2. Antiproliferative Activity of Quinazolin-4(3H)-One Derivative in Human NSCLC Cell Lines

**Figure 2:**
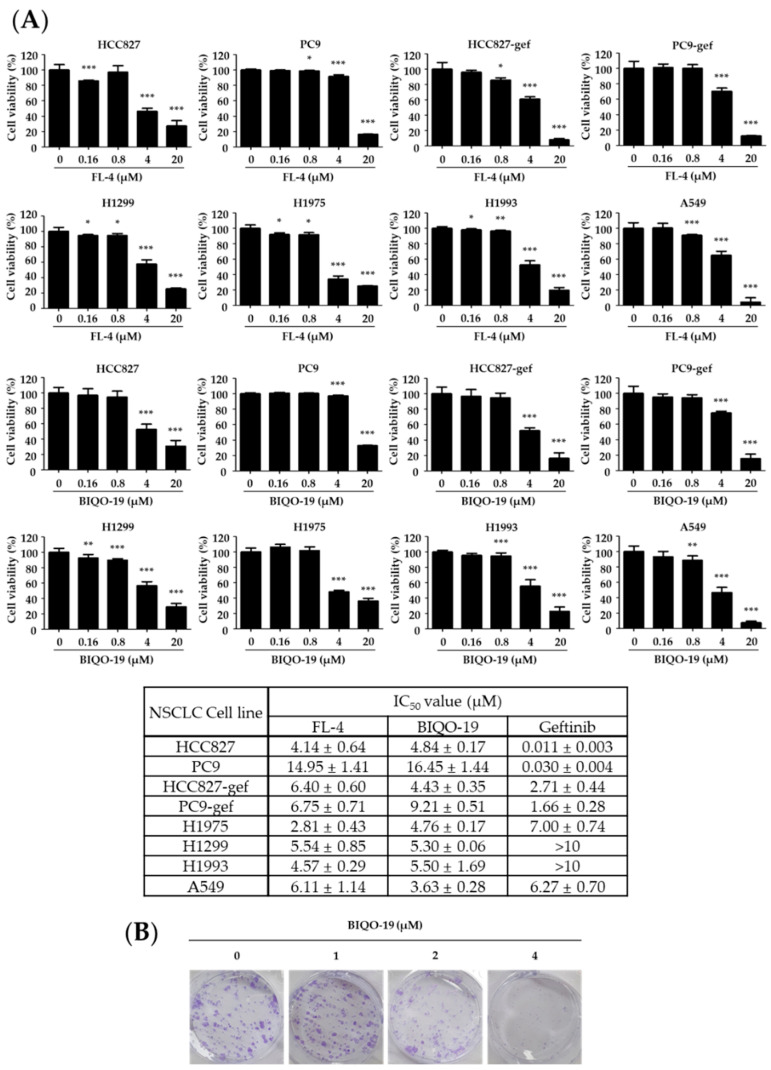
Effects of quinazolin-4(3*H*)-one derivatives on the proliferation of NSCLC cells. (**A**) Diverse NSCLC cell lines were treated with quinazolin-4(3*H*)-one derivatives for 72 h and cell proliferation was measured using a sulforhodamine B (SRB) assay. All data are expressed as the means ± standard deviation (SD) from three independent experiments. * *p* < 0.05, ** *p* < 0.01, and *** *p* < 0.001 indicate statistically significant difference compared with the vehicle-treated control group. The results are summarized in the table as half-maximal concentration (IC_50_) values for the compounds (μM) calculated from the mean of triplicate measurements. (**B**) Effect of BIQO-19 on colony formation in EGFR-TKI-resistant H1975 cells. The cells were treated with various concentrations of BIQO-19 for 48 h, washed, and incubated for an additional ten days. The colonies were fixed and stained with crystal violet.

In order to determine whether quinazolin-4(3*H*)-one derivatives affect the proliferation of NSCLC cells, a sulforhodamine B (SRB) assay was performed to measure the growth inhibition of the test compounds against a variety of NSCLC cell lines. As shown in Figure 2A, the two quinazolin-4(3*H*)-one derivatives, FL-4 and BIQO-19, exhibited antiproliferative activity with an almost similar range of IC_50_ values against NSCLC cells. Interestingly, quinazolin-4(3*H*)-one derivatives effectively inhibited the growth of both the EGFR-TKI-sensitive (HCC827 and PC9) and the EGFR-TKI-resistant (gefitinib resistant cells HCC827-gef, PC9-gef, H1975, H1299, H1993, and A549) cell lines. Based on its activity against the EGFR-TKI-resistant cell lines and improved ADMET profile compared with FL-4, BIQO-19 was selected for further elucidation of its underlying molecular mechanism in EGFR-TKI-resistant cells.

We selected the H1975 cell line, which harbors EGFR, L858R, and T790M double mutations, and is thus considered resistant to gefitinib, an EGFR-TKI. Therefore, identification of the underlying molecular mechanism of BIQO-19 was primarily conducted in the H1975 cells as a representative EGFR-TKI-resistant model. In order to further confirm whether the growth-inhibitory activity of BIQO-19 affects colony formation in H1975 cells, the cells were treated with BIQO-19 for 48 h and were incubated for an additional ten days in the absence of the compound. BIQO-19 exhibited significant inhibition of colony formation in H1975 cells (Figure 2B).

### 2.3. Effects of BIQO-19 on the Activity and Expression of Aurora Kinase A

**Figure 3:**
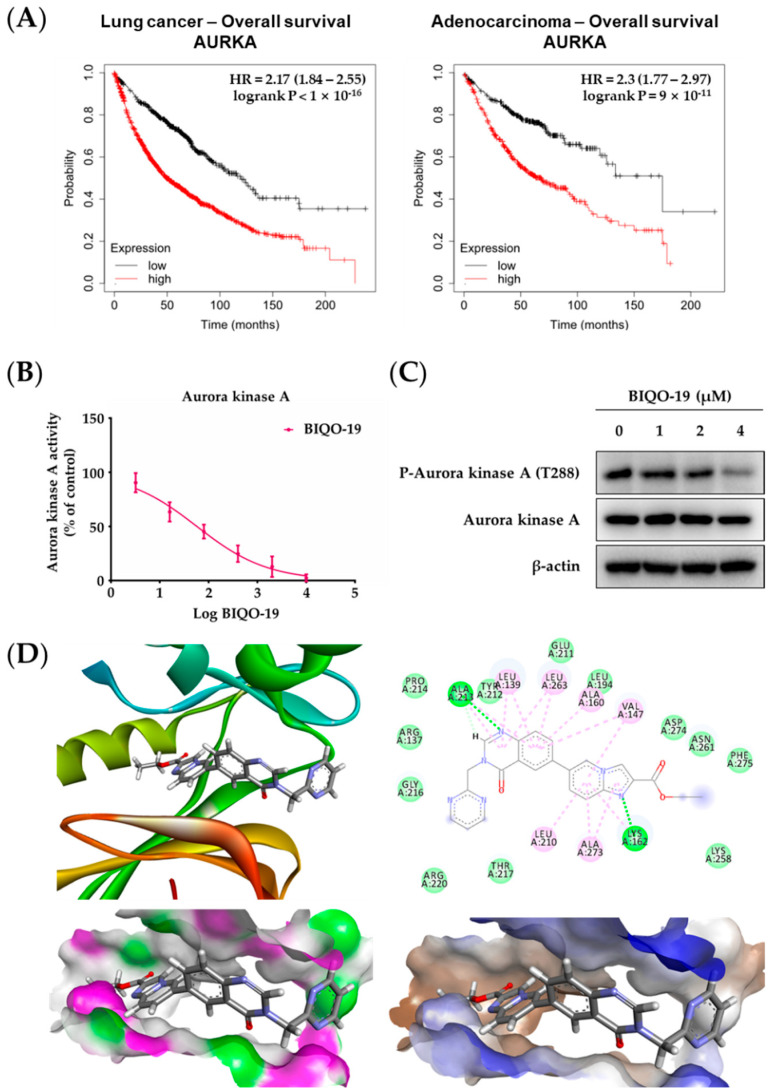
Correlation between expression level of aurora kinase A and overall survival in lung cancer and the effects of BIQO-19 on aurora kinase A activity and expression in H1975 cells. (**A**) The overall survival (OS) curves for lung cancer (left panel) and adenocarcinoma (right panel) with aurora kinase A expression level were analyzed using the Kaplan–Meier method. (**B**) The cell-free enzymatic activity of aurora kinase A was analyzed as described in the Methods section. Data are presented as the means ± standard deviation (SD) from three independent experiments. (**C**) H1975 cells were treated with BIQO-19 for 24 h and the levels of phospho-aurora kinase A (T288) expression were analyzed by Western blotting analysis. β-Actin was used as an internal control. (**D**) Molecular modeling of BIQO-19 and aurora kinase A (PDB ID: 6C2T) binding simulation were carried out in a docking study, as described in the Methods.

In order to confirm a correlation between the expression levels of aurora kinase A and cancer survival, the clinical significance of aurora kinase A expression in patients with lung cancer and adenocarcinoma was analyzed using the Kaplan–Meier method. Patients’ clinical data were split using the auto-select best cutoff method and overall survival (OS) was shown according to aurora kinase A expression levels. Patients with higher expression of aurora kinase A showed significantly decreased OS compared with lower-expression patients in both lung cancer and adenocarcinoma (Figure 3A). These data indicate that aurora kinase A expression is inversely correlated with OS in lung cancer and adenocarcinoma, suggesting that aurora kinase A may be a therapeutic target for the management of NSCLC.

As the expression levels of aurora kinase A are considered to be highly associated with OS in NSCLC, the effects of BIQO-19 on the activity and expression of aurora kinase A were evaluated in H1975 cells. A cell-free kinase assay for aurora kinases revealed that BIQO-19 effectively inhibited the activity of aurora kinase A in a concentration-dependent manner (Figure 3B). In particular, BIQO-19 exhibited a relatively selective inhibition for aurora kinase A (IC_50_; 68.54 nM) compared with aurora kinase B (IC_50_; 581.03 nM) (Table 2). In addition, in order to confirm whether the growth-inhibitory activity of BIQO-19 in H1975 cells was associated with the activation of aurora kinase A, the expression levels of activated-aurora kinase A and phosphorylated aurora kinase A (T288) were evaluated by Western blotting in BIQO-19-treated H1975 cells. As shown in Figure 3C, the levels of p-aurora kinase A (T288) expression were suppressed by BIQO-19 treatment in a concentration-dependent manner without changing total aurora kinase A expression. These data indicate that BIQO-19 effectively inhibits aurora kinase A activity with a selectivity against aurora kinase B and suppresses the activation of aurora kinase A protein expression in H1975 cells. Therefore, BIQO-19 can be considered a potential aurora kinase A inhibitor, and exhibits effective antiproliferative activity against EGFR-TKI-resistant NSCLC cells as well.

To further confirm the effects of BIQO-19 on aurora kinase A activity, a docking study was conducted via molecular simulation. The results indicate that BIQO-19 exhibits good binding affinity for aurora kinase A at its active site. Similar to FL-4, the nitrogen atom of quinazolinone in BIQO-19 forms a conserved hydrogen bond with the amino acid residue Ala213, and the benzyl group at N3 of quinazolinone in BIQO-19 forms van der Waals forces with Thr217, which is a critical residue for aurora kinase-specific inhibition. In addition, the nitrogen atom of imidazopyridine in BIQO-19 forms an additional hydrogen bond with the amino acid residue Lys162, which might contribute to better inhibitory activity of BIQO-19 compared to its lead compound, Fl-4. Similar to the binding model of FL-4, the fragments of quinazolinone and imidazopyridine form hydrophobic interactions, including Pi–Alkyl interactions, with the residues Leu139, Val147, Ala160, Leu210, and Ala273, and van der Waals with Agr137, Leu194, Leu212, Pro214, Gly216, Thr217, Lys218, Arg220, Asn261, and Asp274 (Figure 3D). MOE provides the docking score as the S value; the lower the S value, the stronger the binding affinity. More importantly, the S value (docking score: −9.438) of BIQO-19 is lower than that of FL-4 (−8.217) and of the crystallization ligand (−6.815), indicating its better binding affinity with aurora kinase A. The molecular docking study thus revealed the potential inhibitory activity of BIQO-19 against aurora kinase A activity.

### 2.4. Effects of BIQO-19 on Cell Cycle Regulation in H1975 Cells

**Figure 4:**
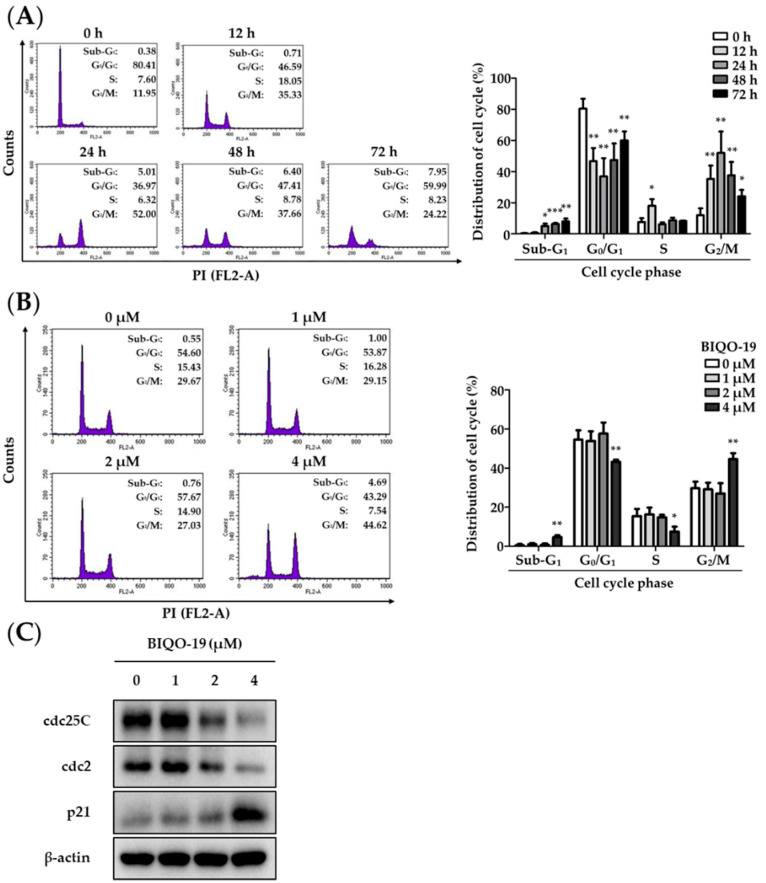
Effects of BIQO-19 on cell cycle distribution in H1975 cells. (**A**) Cells were treated with BIQO-19 (4 μM) for the indicated time points (0–72 h), collected, fixed, and stained with propidium iodide (PI). The cell cycle distribution was analyzed by flow cytometry. Data are presented as the means ± SD from three independent experiments. * *p* < 0.05, ** *p* < 0.01, and *** *p* < 0.001 indicate statistically significant difference compared with the vehicle-treated control group. (**B**) Cells were treated with various concentrations of BIQO-19 for 24 h, collected, and fixed. The cells were stained with PI and the cell cycle distribution was analyzed by flow cytometry. Data are representative of three independent experiments and are presented as the means ± SD from three independent experiments. * *p* < 0.05 and ** *p* < 0.01 indicate a statistically significant difference compared with the vehicle-treated control group. (**C**) Cells were treated with various concentrations of BIQO-19 for 24 h and the expression levels of cdc25C, cdc2, and p21 were evaluated by Western blotting analysis. β-Actin was used as an internal control.

As aurora kinase A plays a major role in the regulation of mitosis, we determined whether the antiproliferative activity of BIQO-19, an aurora kinase A inhibitor, was associated with cell cycle arrest during mitosis. The distribution of cell populations was analyzed by flow cytometry after treatment with BIQO-19 for up to 72 h in H1975 cells. As shown in Figure 4A, treatment with BIQO-19 (4 μM) for the indicated times increased the population of cells in the G_2_/M phase from 12.0% (0 h), 35.3% (12 h), and up to 52.0% (24 h). Subsequently, longer treatment (>24 h) with BIQO-19 decreased the G_2_/M phase cell population; however, it increased the sub-G_1_ phase cell population in a time-dependent manner. The increase in the G_2_/M phase after treatment with BIQO-19 for 24 h occurred in a concentration-dependent manner (Figure 4B). It is known that cell division cycle 25c (cdc25c) and cyclin-dependent kinase cdc2 play a crucial role in the regulation of the G_2_/M phase [43]. In addition, cell cycle inhibitor p21 participates in cell cycle arrest by inhibiting cyclin dependent kinases [44]. In order to further confirm whether the effect of BIQO-19 on the G_2_/M cell cycle arrest is associated with the expression of cell cycle regulators, the expressions of the cdc25C, cdc2, and p21 proteins were analyzed by Western blotting after treatment of H1975 cells with BIQO-19 for 24 h. As shown in Figure 4C, BIQO-19 effectively downregulated cdc25C and cdc2 expression and upregulated p21 expression (Figure 4C). These data suggest that the antiproliferative activity of BIQO-19 is in part associated with the induction of G_2_/M phase cell cycle arrest.

### 2.5. Effects of BIQO-19 on the Induction of Apoptosis in H1975 Cells

**Figure 5:**
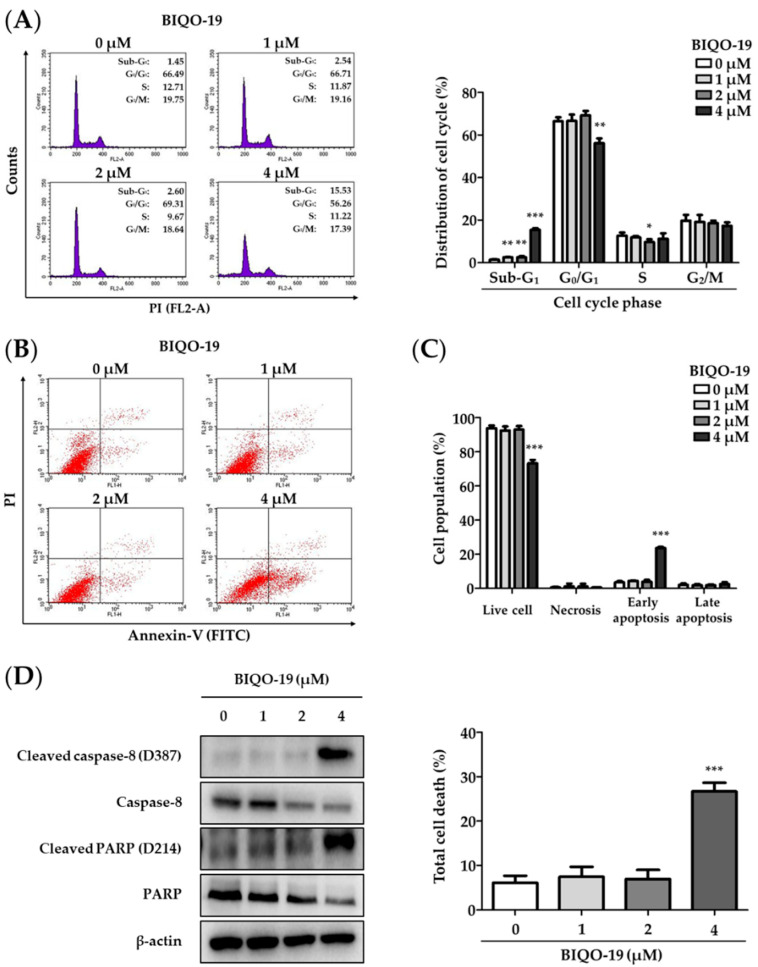
Effects of BIQO-19 on the induction of apoptosis in H1975 cells. (**A**) Cells were treated with various concentrations of BIQO-19 for 72 h, collected, fixed, and stained with PI. The cell cycle distribution was analyzed by flow cytometry. Histograms are presented as a representative of three independent experiments (left panel) and data are presented as the means ± SD (*n* = 3). * *p* < 0.05, ** *p* < 0.01, and *** *p* < 0.001 indicate a statistically significant difference compared with the vehicle-treated control group (right panel). (**B**,**C**) Cells were treated with BIQO-19 for 72 h and stained with Annexin V-fluorescein isothiocyanate (FITC) and PI. After staining, cell populations with Annexin V/FITC (+) and/or PI (+) were analyzed by flow cytometry (**B**). The percentage of each cell population (live, necrosis, early apoptosis, late apoptosis) (**C**, upper panel) and the percentage of total cell death (early apoptosis + late apoptosis + necrosis) were quantified and displayed as % of control (**C**, bottom panel). Data were obtained from three independent experiments and are presented as the means ± SD. *** *p* < 0.001 indicates a statistically significant difference compared with the vehicle-treated control group. (**D**) Cells were treated with various concentrations of BIQO-19 for 72 h and the expression levels of cleaved caspase-8, caspase-8, cleaved PARP, and PARP were analyzed by Western blotting analysis. β-Actin was used as an internal control.

As we observed an increase in the sub-G_1_ phase cell population after longer treatment with BIQO-19 (4 μM) for 72 h (Figure 3A), further study was designed in order to examine whether long-term treatment with BIQO-19 induced apoptotic cell death. H1975 cells were treated with various concentrations of BIQO-19 for 72 h and the sub-G_1_ phase cell population, which represents the apoptotic events with DNA fragmentation, was analyzed by flow cytometry. As shown in Figure 5A, the cell populations in the sub-G_1_ phase were increased into 2.54%, 2.60%, and 15.5% after treatment with 1, 2, and 4 μM of BIQO-19, respectively, in H1975 cells. These data indicate that long-term treatment with BIQO-19 induces apoptotic cell death in H1975 cells. In addition, the induction of apoptosis after BIQO-19 treatment was supported by flow cytometry using cells double stained with Annexin V-FITC and propidium iodide (PI). Treatment with 4 μM BIQO-19 for 72 h effectively increased the apoptotic cell populations, including those in the early and late apoptosis stages, with 26.67% of total cell deaths in H1975 cells (Figure 5B,C).

To further confirm whether the induction of apoptosis by BIQO-19 is associated with the expression of apoptosis-related proteins, the expressions of cleaved PARP, PARP, cleaved caspase-8, and caspase-8 proteins were analyzed by Western blotting analysis after treatment of H1975 cells with BIQO-19 for 72 h. BIQO-19 treatment upregulated the expression levels of cleaved PARP and cleaved caspase-8 (activated forms) and downregulated the expression levels of PARP and caspase-8 expression (inactivated forms) in a concentration-dependent manner (Figure 5D). Together, these findings indicate that prolonged exposure of H1975 cells to BIQO-19 results in the induction of apoptotic cell death.

### 2.6. Combination Effects of BIQO-19 and Gefitinib on the Proliferation of H1975 Cells

**Figure 6:**
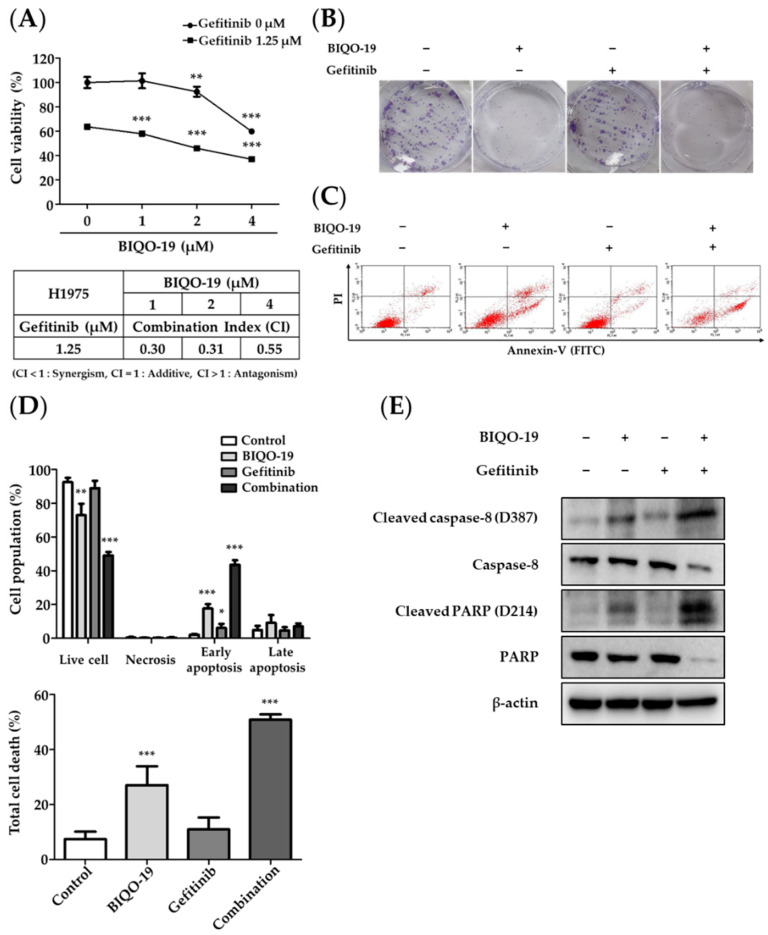
Effects of a combination of BIQO-19 and gefitinib on the proliferation of H1975 cells. (**A**) Cells were treated with BIQO-19 and gefitinib for 48 h and the proliferation of the cells was evaluated using SRB assay. Data are presented as means ± SD (*n* = 3). ** *p* < 0.01 and *** *p* < 0.001 indicate statistically significant difference compared to the vehicle-treated control group. (**B**) Effect of combined BIQO-19 and gefitinib treatment on colony formation in EGFR-TKI-resistant H1975 cells. The cells were treated with BIQO-19 and gefitinib for 48 h, washed, and incubated for an additional 1ten0 days. The colonies were fixed and stained with crystal violet. (**C**,**D**) Cells were treated with BIQO-19 and gefitinib for 72 h and stained with Annexin V-FITC and PI. After staining, the cell populations with Annexin V/FITC (+) and/or PI (+) were analyzed by flow cytometry (**C**). The percentage of each cell population (live, necrosis, early apoptosis, late apoptosis) (**D**, upper panel) and the percentage of total cell death (early apoptosis + late apoptosis + necrosis) were quantified and presented as % of control (**D**, bottom panel). Data were obtained from three independent experiments and are presented as the means ± SD. * *p* < 0.05, ** *p* < 0.01, and *** *p* < 0.001 indicate a statistically significant difference compared with the vehicle-treated control group. (**E**) Cells were treated with BIQO-19 and gefitinib for 72 h and the expression levels of cleaved caspase-8, caspase-8, cleaved PARP, and PARP were evaluated by Western blotting analysis. β-Actin was used as an internal control.

Overcoming EGFR-TKI resistance is one of the most practical issues facing the successful treatment of patients with NSCLC. Many studies have addressed the restoration of gefitinib sensitivity in EGFR-TKI-resistant NSCLC cells [45,46]. Herein, we investigated whether the aurora kinase A inhibitor BIQO-19 was able to resensitize EGFR-TKI-resistant H1975 cells to gefitinib. The combination of BIQO-19 and gefitinib exerted higher antiproliferative activity compared with single-agent treatment; the synergistic effects were determined by calculating a CI value using the Chou–Talalay method (CI < 1) (Figure 6A). The combination of BIQO-19 with gefitinib effectively inhibited colony formation in H1975 cells compared with single-agent treatment (Figure 6B). Flow cytometry analysis with Annexin V-FITC and PI double staining of cells after treatment with a combination of BIQO-19 and gefitinib for 72 h revealed synergistic effects. The early apoptosis fraction exhibited an increase from 17.6% with BIQO-19 single treatment to 43.5% with the combination of BIQO-19 and gefitinib treatment (Figure 6C). Additionally, total cell death was increased from 27.1% with BIQO-19 single treatment to 50.9% with the combination of BIQO-19 and gefitinib treatment (Figure 6D). These synergistic effects of the combination were confirmed by the observation of upregulated apoptotic biomarker expression, including cleaved PARP and cleaved caspase-8, in H1975 cells (Figure 6E). These findings indicate that BIQO-19 re-sensitizes EGFR-TKI-resistant NSCLC cells to gefitinib; combined treatment with BIQO-19 and gefitinib synergistically inhibits cell proliferation and induces apoptosis in H1975 cells.

## 3. Discussion

Although several important findings associated with new strategies to treat lung cancer have been discovered over last two decades, lung cancer remains the leading cause of cancer deaths globally. In particular, epidermal growth factor receptor-tyrosine kinase inhibitors (EGFR-TKIs) have been developed as targeted agents for patients with lung cancers harboring EGFR mutations. Initially, the response rates for these patients increased. However, treatment with EGFR-TKIs for several months eventually results in acquired resistance to these agents [8]. Recently, immunotherapy has been introduced and represents a promising therapy for treating diverse cancers. PD-L1 inhibitors were approved as first-line therapy for patients with PD-L1-positive metastatic NSCLC in 2016. However, low response rates were observed in patients with EGFR-TKI resistance [47,48], suggesting that the introduction of immunotherapy could not fully resolve the problem of EGFR-TKI resistance. Currently, no effective therapeutic options for patients with EGFR-TKI-resistant NSCLC exist. Therefore, identifying new therapeutic approaches to overcome EGFR-TKI resistance in NSCLC is necessary.

The cell cycle is tightly regulated in mammalian cells. Dysregulation of the cell cycle machinery is a driving force in lung cancer malignancy, and thus targeting cell cycle regulatory proteins is considered an effective way to suppress lung cancer proliferation [49]. Aurora kinase A is a major cell cycle regulatory protein which is overexpressed in cancer cells [14,18]. We analyzed the expression of aurora kinase A and discovered a negative correlation between expression levels and OS rates in patients with lung cancer and adenocarcinoma (a major representative form of NSCLC). Moreover, patients with lung cancer and NSCLC exhibiting higher expression of aurora kinase A demonstrated relatively lower OS rates. These findings indicate that targeting aurora kinase A is a clinically relevant approach for treating patients with lung cancer and adenocarcinoma. In addition, the association of aurora kinase A with drug resistance has been reported in several cancers, including gastric cancer, prostate cancer, and breast cancer [50,51,52]. In particular, studies have revealed that acquired resistance to EGFR-TKIs in NSCLC cells requires aurora kinase A activity and that EGFR-TKI-resistant NSCLC cells are sensitive to aurora kinase inhibitors [26]. Therefore, targeting aurora kinase A represents a promising strategy to inhibit the growth of EGFR-TKI-resistant NSCLC cells.

As part our continuous efforts to identify novel anticancer agents, we previously reported the development of aurora kinase inhibitors as candidate anticancer agents [42,53]. The designed quinazolin-4(3*H*)-one derivatives, including FL-4, were previously shown to exhibit aurora kinase inhibitory activity and antiproliferative effects on triple-negative breast cancer cell lines [42]. In the present study, we newly designed and synthesized BIQO-19, a FL-4 derivative, in order to develop a better druggable candidate compound with enhanced pharmacokinetic properties. BIQO-19 was designed to exhibit enhanced solubility thanks to the replacement of tryptamine with pyrimidine and the addition of an ester group to FL-4, which we expected to increase polarity [54]. Initially, pharmacokinetic profiling of quinazolin-4(3*H*)-one derivatives was predicted via computational analysis (Discovery Studio 3.0 software package). The pharmacokinetic profiles of the ADMET levels revealed higher values for BIQO-19 compared with those of FL-4, indicating that BIQO-19 might be more suitable as a druggable candidate from the point of view of pharmacokinetics. The antiproliferative activity of quinazolin-4(3*H*)-one derivatives was evaluated in various lung cancer cell lines, including EGFR-TKI-resistant NSCLC cell lines. BIQO-19 exhibited broad and effective antiproliferative activity against all tested lung cancer cell lines, including EGFR-TKI-resistant NSCLC cells. As BIQO-19 possesses an enhanced ADMET profile and effective antiproliferative activity in EGFR-TKI-resistant NSCLC cells, further studies were performed in order to elucidate its molecular mechanism of action in EGFR-TKI-resistant H1975 cells. We found that BIQO-19 effectively and selectively inhibited aurora kinase A activity compared with aurora kinase B activity in a cell-free enzyme assay. In addition, BIQO-19 effectively suppressed the activation of aurora kinase A in H1975 cells. These findings confirm that the antiproliferation activity of BIQO-19 in H1975 cells is partly associated with the regulation of aurora kinase activation in cancer cells. The main function of aurora kinase A is to regulate the cell cycle during mitosis. In the present study, we found that BIQO-19 effectively inhibited the activation of aurora kinase A, which resulted in cell cycle arrest in the G_2_/M phase at relatively short compound exposure times, and subsequently induced apoptosis following long-term treatment in H1975 cells. These findings suggest that the antiproliferative activity of BIQO-19 is associated with effective inhibition of aurora kinase A, which in turn leads to the induction of G_2_/M phase cell cycle arrest and apoptotic cell death in EGFR-TKI-resistant NSCLC cells. Recently, diverse aurora kinase inhibitors have been developed and reported to exhibit antiproliferative activity in many cancer types, and several aurora kinase inhibitors have entered clinical trials [55,56]. Although aurora kinase inhibitors alone do not show increased effects compared with commercially used chemotherapy or targeted therapies, combination therapy using aurora kinase inhibitors with standard therapies results in a significant improvement in patient survival [57]. Therefore, we evaluated the combination of BIQO-19 and gefitinib, an EGFR-TKI, on the proliferation of EGFR-TKI-resistant NSCLC cells. We found that the combination of BIQO-19 and gefitinib enhanced antiproliferative activity. These findings indicate that the use of an aurora kinase A inhibitor in combination with gefitinib is an attractive strategy for treating patients with EGFR-TKI-resistant NSCLC. However, elucidating the detailed mechanisms of these effects requires further study.

## 4. Materials and Methods

### 4.1. Chemistry

All reagents and solvents were commercially available and used without further purification. ^1^H-NMR and ^13^C-NMR spectra were recorded on a Bruker AVANCE NEO 600 MHz NMR spectrometer, and the NMR spectra were generated using Mestrenova 12.0 processing software. Spectra were recorded at 25 °C using dimethyl sulfoxide-*d*_6_ or methanol-*d*_4_ as solvents, with tetramethylsilane (TMS) as an internal standard. All chemical shifts are reported in ppm (*δ*) and coupling constants (*J*) are reported in hertz (Hz). All melting points were determined on a Beijing micro-melting-point apparatus, and the values were uncorrected. High-resolution exact mass measurements were performed using electrospray ionization (positive mode) on a quadrupole time-of-flight (QTOF) mass spectrometer (microTOF-Q, Bruker Inc., Billerica, MA, USA). Column chromatography was performed on silica gel (Qingdao, China) using the indicated eluents. Thin-layer (0.25 mm, GF254) chromatography was carried out on silica gel plates (Qingdao, China).

#### General Procedure for the Synthesis of Ethyl 6-(4-Oxo-3-(pyrimidin-2-ylmethyl)-3,4-dihydroquinazolin-6-yl)imidazo [1,2-a]pyridine-2-carboxylate (BIQO-19)

The synthesis of the compound BIQO-19 is summarized in Scheme 1. First, 2-amino-5-bromo benzoic acid (**1**) and pyrimidin-2-ylmethanamine (**2**) were used as starting materials to prepare 6-bromo-3-(pyrimidin-2-ylmethyl)quinazolin-4(3*H*)-one (**3**). Step 1: A mixture of **1** (5 mmol, 10.75 g), **2** (7.5 mmol, 7.18 mg), triethoxymethane (7.5 mmol, 11.10 g), and I_2_ (0.05 mmol, 0.13 g) were suspended in ethanol (EtOH) (150 mL) under a nitrogen atmosphere at 25 °C. The reaction mixture was heated to 80 °C and stirred for 6 h until reaction completion was confirmed by thin-layer chromatography (TLC). The reaction mixture was cooled to room temperature and the ethanol vaporized under reduced pressure. The mixture was further dissolved with 200 mL ethyl acetate and washed once with 1 M sodium hydroxide solution. The sodium hydroxide solution was extracted with ethyl acetate three times, combined with ethyl acetate extract, and washed successively with 1 M NaOH and saturated salt water. The ethyl acetate extract was collected and dried with anhydrous sodium sulfate overnight. Sodium sulfate was removed by filtration and the ethyl acetate extract was concentrated to obtain compound **3** (6-bromo-3-(pyrimidin-2-ylmethyl)quinazolin-4(3*H*)-one). Step 2: Compound **3** (10 mmol, 3.17 g), compound **4** (pinacol ester, 15 mmol, 3.3 g) and PdCl_2_(dppf) (0.28 mmol, 0.21 g) in anhydrous 1,4-dioxane (30 mL) was stirred under argon at 100 °C for 4 h until reaction completion, then confirmed by TLC analysis. 1,4-Dioxane was removed under reduced pressure and the mixture was diluted with water (100 mL), then the mixture was extracted with ethyl acetate (3 × 100 mL). The organic layer was collected and washed with water (2 × 100 mL), dried with Na_2_SO_4_ and evaporated to obtain a yellow solid (**5**). Step 3: Compound **5** (10 mmol, 330 mg) and compound **6** (ethyl bromopyruvate, 30 mmol, 540 mg) were added into the EtOH (60 mL) under a nitrogen atmosphere. The reaction mixture was stirred at 80 °C for 2 h and cooled to 25 °C. NaHCO_3_ (30 mmol, 2.52 g) was added to the reaction mixture and stirred again for 12 h at 80 °C until reaction completion. The reaction mixture was cooled to 25 °C and the EtOH was removed under reduced pressure. The resulting products were purified using silica gel chromatography. Compound **3**, 6-bromo-3-(pyrimidin-2-ylmethyl)quinazolin-4(3H)-one, was obtained as a white solid, yield 73.4%, ESI-MS: m/z 339.1, 341.1 [M + Na]^+^. Compound **5**, 6-(6-aminopyridin-3-yl)-3-(pyrimidin-2-ylmethyl)quinazolin-4(3H)-one, was obtained as a white solid, yield 83.4%, ESI-MS: m/z 331.1 [M + H]^+^. BIQO-19 was separated as white crystals, yield 87.4%, m.p. 284–286 °C. ^1^H-NMR (600 MHz, CDCl_3_) δ 8.68 (d, *J* = 4.9 Hz, 2H), 8.49 (d, *J* = 2.2 Hz, 1H), 8.41 (d, *J* = 1.8Hz, 1H), 8.24 (s, 1H), 8.21 (s, 1H), 7.96 (dd, *J* = 2.2, 8.4 Hz, 1H), 7.86 (d, *J* = 8.4 Hz, 1H), 7.76 (d, *J* = 9.4 Hz, 1H), 7.57 (dd, *J* = 1.8 Hz, 9.4 Hz, 1H), 7.23 (t, *J* = 4.9 Hz, 9.4Hz, 1H), 5.45 (s, 2H), 4.46 (q, *J* =7.1, 2H), 1.44 (t, *J* = 7.1 Hz, 3H). ^13^C-NMR (151 MHz, CDCl_3_) δ 164.50, 163.17, 161.06, 157.58, 148.15, 147.78, 144.54, 137.61, 135.54, 132.70, 128.76, 126.98, 126.59, 125.0, 123.8, 122.9, 120.2, 119.3, 117.6, 61.4, 51.7, 14.5. HRMS (ESI-QTOF) m/z Calcd. For C_23_H_19_O_3_N_6_ [M + Na]^+^ m/z: 449.1333, found: 449.1324. NMR and high resolution mass spectras are shown in Figures S1–S3.

### 4.2. Reagents

Culture media, fetal bovine serum, antibiotic-antimycotic solution (penicillin 10,000 units/mL, streptomycin 10,000 µg/mL, amphotericin B 25 µg/mL), and trypsin-EDTA solution (1×) were purchased from Gibco (Grand Island, NY, USA). Gefitinib (CAS No. HY-50895) was purchased from MedChemExpress (Monmouth Junction, NJ, USA). Trichloroacetic acid (TCA), sulforhodamine B (SRB), crystal violet solution, and bovine serum albumin (BSA) were purchased from Sigma-Aldrich (St. Louis, MO, USA). Anti-aurora kinase A (#14475), anti-p-aurora kinase A (T288) (#3079), anti-p21 Waf1/Cip1 (#2947), anti-cdc2 (#9116), anti-cdc25C (#4688), anti-PARP (#9532), anti-Cleaved PARP (D214) (#5625), anti-Caspase-8 (#9746), and anti-Cleaved Caspase-8 (D374) (#9496) antibodies were obtained from Cell Signaling Technology (Danvers, MA, USA).

### 4.3. ADMET Prediction Analysis

The ADMET properties of the FL-4 and BIQO-19 compounds were calculated with the ADMET descriptors of Discovery Studio 3.0. The ADMET descriptors parameter was set as the default. Each of the ADMET profile levels were categorized by following the criteria. ADMET_Solubility_Level: 0 (extremely low, log (Sw) < −8.0), 1 (very low, −8.0 < log (Sw) < −6.0), 2 (low, −6.0 < log (Sw) < −4.1), 3 (good, −4.1 < log (Sw) < −2.0), 4 (optimal, −2.0 < log (Sw) ≤ 0.0), 5 (too soluble, 0.0 < log (Sw)), 6 (warning: molecules with one or more unknown AlogP98 types). ADMET_BBB_Level: 0 (very high penetrant, logBB ≥ 0.7), 1 (high penetrant, 0 ≤ logBB < 0.7), 2 (medium penetrant, −0.52 < logBB < 0), 3 (low penetrant, logBB ≤ −0.52), 4 (undefined).

### 4.4. Cell Culture

The human non-small lung cancer cell lines A549, NCI-H1975, NCI-H1299, and NCI-H1993 were purchased from the American Type Culture Collection (Manassas, VA, USA). The human non-small cell lung cancer cell lines HCC827, PC9, HCC827-gef and PC9-gef were a kind gift from Dr. Jae Cheol Lee and Dr. Jin Kyung Rho (Asan Medical Center, Seoul, Korea). HCC827-gef and PC9-gef cells were subcultured in the presence of 1 µM gefitinib. All cells were cultured in RPMI 1640 medium supplemented with 10% fetal bovine serum and 1% antibiotic-antimycotic. Cells were incubated at 37 °C in a humidified atmosphere with 5% CO_2_.

### 4.5. Cell Proliferation Assay

Cell viability was measured using the SRB method as described previously [58]. Briefly, cells were seeded with different concentrations of compounds and incubated for 72 h. After 72 h incubation, cells were fixed with 10% TCA for 30 min at 4 °C and stained with 0.4% (*w*/*v*) SRB in 1% acetic acid for 2 h at room temperature. The stained proteins were dissolved in 10 mM Tris solution (pH 10) and measured the absorbance at 515 nm with a VersaMax ELISA microplate reader (Molecular Devices, Sunnyvale, CA, USA).

### 4.6. Colony Formation Assay

Cells (500 cells/well) were seeded and cultured overnight. The compounds were treated for 48 h, the cells were washed with phosphate buffered saline (PBS), and continued incubation. Culture medium was replaced every three days for ten days. Cells were fixed with 4% paraformaldehyde solution and stained with 0.1% crystal violet.

### 4.7. Bioinformatics Analysis

The OS of patients with lung cancer and adenocarcinoma with different expression levels of Aurora Kinase A was analyzed through a Web-based meta-analysis Kaplan–Meier plotter (http://www.kmplot.com/lung, accessed on 6 April 2022). A total of 1925 lung cancer patient gene expression values and survival data were analyzed using multiple microarray datasets [59].

### 4.8. Aurora A Kinase Assay

Aurora A kinase activity was measured using an ADP-Glo™ Kinase assay kit (Promega, USA, Catalog No. # PAV9101). Recombinant human aurora A protein (Active) (ab271368) and Recombinant human aurora B protein (Active) (ab51435) were purchased from Abcam (United States, Boston, MA). Myelin basic protein was selected as a substrate and staurosporine was used as a positive control. The ADP-Glo ^TM^ reagents and kinase detection buffer and substrates were prepared according to the ADP-Glo™ Kinase Assay kit instructions. Luminescence was measured using a microplate reader.

### 4.9. Western Blotting Analysis

The cells were collected and lysed in sample loading buffer (Bio-Rad, Hercules, CA, USA). Total protein concentrations were quantified using a BCA Protein Assay Kit (Thermo Fisher Scientific, Waltham, MA, USA). Quantified proteins were loaded onto SDS-polyacrylamide gels and separated by molecular weight via electrophoresis (PAGE). Then, proteins were transferred to polyvinylidene fluoride membranes (Millipore, Bedford, MA, USA); 5% BSA in Tris-buffered saline containing 0.1% Tween-20 (TBST) was used for blocking. The membranes were blocked for 30 min at room temperature with mild shaking, then incubated with antibodies diluted in 2.5% BSA in TBST overnight at 4 °C with mild shaking. Following secondary antibody incubation and washing with TBST, the chemiluminescence signals were detected using the WEST-Queen detection system (iNtRON Biotechnology, Seongnam, Korea) and LAS-4000 (Fuji Film Corp., Tokyo, Japan).

### 4.10. Molecular Docking

A molecular simulation was carried out using the Molecular Operating Environment (MOE, version 2020). BIQO-19 was built in MOE using the Builder program and energy-minimized for docking simulation. The 3D structure of aurora A (PDB ID: 6C2T) was obtain from the PDB databank and optimized, 3-D protonated, and energy-minimized using the default parameters of MOE. The docking site was defined by the Site Finder application, including the residues within 10 Å of the cocrystallization ligand. The docking results were analyzed using Accelrys Discovery Studio Visualizer 4.5.

### 4.11. Cell Cycle Analysis

Cells were seeded and starved in serum-free medium overnight. After starvation, the cells were treated with compounds for the indicated times. After incubation, cells were harvested, washed with PBS, and fixed with 70% ethanol in PBS overnight at −20 °C. The fixed cells were washed with PBS, resuspended in 100 µg/mL of RNase A for 30 min at room temperature, and stained with 50 µg/mL PI. Fluorescence intensity was analyzed using a FACSCalibur flow cytometer (BD Biosciences, San Jose, CA, USA) and BD CellQuest^TM^ Pro version 6.0 software (BD Biosciences, San Jose, CA, USA).

### 4.12. Annexin V-FITC/PI Double Staining

Cells were treated with samples for 72 h and stained with an Annexin V-FITC apoptosis detection kit (BD Biosciences, San Jose, CA, USA) according to the manufacturer’s instructions. Briefly, cells were collected after 72 h treatment, washed with PBS, resuspended in 1× binding buffer, and stained with Annexin V-FITC and PI in the dark for 15 min. After incubation, the cells were diluted with 1× binding buffer and analyzed using an FACSCalibur flow cytometer (BD Biosciences, San Jose, CA, USA) and BD CellQuest^TM^ Pro version 6.0 software (BD Biosciences, San Jose, CA, USA).

### 4.13. Statistical Analysis

Data were obtained from three independent experiments, and are shown as the means ± standard deviation. Statistical significance (* *p* < 0.05, ** *p* < 0.01, *** *p* < 0.001) was calculated using one-way analysis of variance coupled with Dunnett’s *t*-test or Student’s *t*-test.

## 5. Conclusions

In conclusion, we newly introduced a quinazolin-4(3H)-one derivative, BIQO-19, which is an aurora kinase A inhibitor that exhibits an improved ADMET profile and antiproliferative activity in NSCLC cells. The mechanisms of action for the antiproliferative activities of BIQO-19 in EGFR-TKI-resistant NSCLC H1975 cells involve aurora kinase A-mediated arrest at the G2/M phase of the cell cycle and the induction of apoptosis. Combined treatment with BIQO-19 and gefitinib exhibits synergetic activity with respect to inhibiting proliferation and inducing apoptosis in H1975 cells. Therefore, targeting aurora kinase A using a new class of inhibitors in combination with EGFR-TKIs represents a potential therapeutic strategy for treating patients with EGFR-TKI-resistant NSCLC.

## Figures and Tables

**Table 1:** Pharmacokinetic profiles of FL-4 and BIQO-19 as determined by in silico prediction.

Property ^a^	FL-4	BIQO-19
ADMET_Solubility ^b^	−6.224	−3.318
ADMET_Solubility_Level	1 (Very low solubility)	3 (Good solubility)
ADMET_PSA_2D ^c^	105.01	97.34
ADMET_AlogP98 ^d^	4.522	1.802
ADMET_BBB ^e^	Undefined	−1.137
ADMET_BBB_Level	4 (Undefined)	3 (Low penetration)
ADMET_EXT_CYP2D6 ^f^	−9.26	−7.69
ADMET_EXT_hepatotoxic ^g^	4.1775	−3.179
ADMET_EXT_PPB ^h^	3.54856	−0.242418

^a^ Pharmacokinetic properties are categorized as described in the Methods section. ^b^ ADMET_Solubility [log(sw)]: the base 10 logarithm of the molar solubility as predicted by regression. ^c^ ADMET_PSA_2D: the polar surface area value, which is closely related to the absorption properties of compounds. ^d^ ADMET_AlogP98: the logarithm of the partition coefficient between n-octanol and water. ^e^ ADMET_BBB [logBB]: the base 10 logarithm of (brain concentration)/(blood concentration) as predicted by a robust (least-median-of-squares) regression derived from published in vivo brain penetration data. ^f^ ADMET_EXT_CYP2D6: the CYP2D6 enzyme inhibition value. ^g^ ADMET_EXT_hepatotoxic: the potential liver toxicity, classified using the cutoff Bayesian score of −4.154 obtained by minimizing the total number of false positives and false negatives. ^h^ ADMET_EXT_PPB: the plasma protein binding value.

**Table 2:** Effect of BIQO-19 on the enzyme activity of aurora kinases.

Compound	Aurora Kinase A Inhibition (IC_50_) ^a^	Aurora Kinase B Inhibition (IC_50_) ^a^
BIQO-19	68.54 ± 6.45 nM	581.03 ± 13.77 nM
Staurosporine ^b^	18.09 ± 2.07 nM	26.13 ± 2.51 nM

^a^ Results are expressed as the calculated half-maximal concentration (IC_50_) values of the compounds (nM) from the mean of triplicate measurements. ^b^ Staurosporine was used as a positive control.

## Data Availability

Data is contained within the article and supplementary files.

## Supplementary Materials

The following supporting information can be downloaded at: https://www.mdpi.com/article/10.3390/ph15060698/s1, Figure S1: ^1^H-NMR spectra; Figure S2: ^13^C-NMR spectra; Figure S3: High resolution mass spectra.
