# Supplementary material for: Antiproliferative Activity of a New Quinazolin-4(3H)-One Derivative via Targeting Aurora Kinase A in Non-Small Cell Lung Cancer
Source: Pharmaceuticals (Basel). 2022 Jun 2;15(6):698. doi: 10.3390/ph15060698 (PMC9228987; doi:10.3390/ph15060698)
Supplement: Supplementary file 1 [file pharmaceuticals-15-00698-s001.zip › pharmaceuticals-1727485-supplementary.pdf]

*Communication*

# Antiproliferative Activity of a New Quinazolin-4(3*H*)-One Derivative via Targeting Aurora Kinase A in Non-Small Cell Lung Cancer

Ji Yun Lee [1], Huarong Yang [2], Donghwa Kim [1], Kay Zin Kyaw [1], Ruoci Hu [1], Yanhua Fan [2,3,*] and Sang Kook Lee [1,*]

[1] College of Pharmacy, Natural Products Research Institute, Seoul National University, Seoul 08826, Korea; jiyunkr0@naver.com (J.Y.L.); dkim0719@snu.ac.kr (D.K.); dawkayzinkyaw@gmail.com (K.Z.K.); 18502407040@163.com (R.H.)

[2] The Key Laboratory of Chemistry for Natural Products of Guizhou Province and Chinese Academy of Sciences, Guiyang 550014, China; 2018087@stu.gzy.edu.cn

[3] State Key Laboratory for Functions and Applications of Medicinal Plants, Guizhou Medical University, Guiyang 550014, China

* Correspondence: yhfan@gzcnp.cn (Y.F.); sklee61@snu.ac.kr (S.K.L.); Tel.: +82-2-880-2475 (S.K.L.)

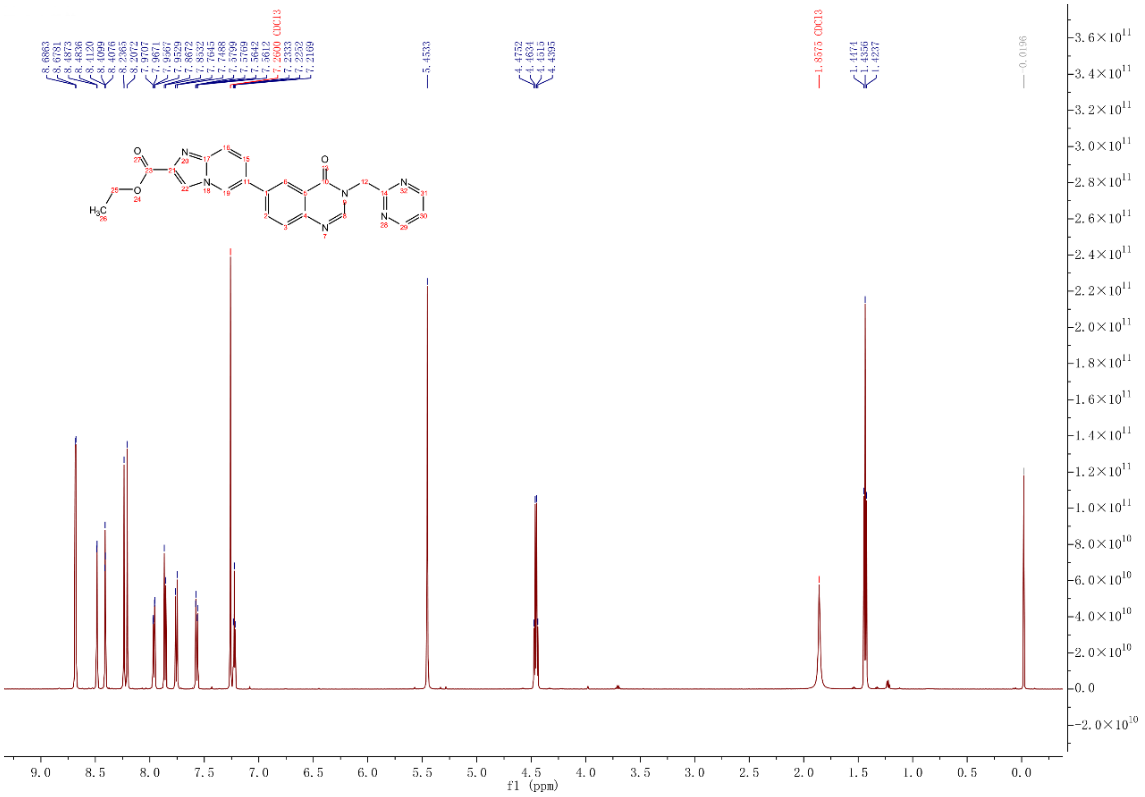


**Figure S1. $^{1}$H-NMR spectra**

$^{1}$H-NMR (600 MHz, $CDCl_3$) δ 8.68 (d, *J* = 4.9 Hz, 2H), 8.49 (d, *J* = 2.2 Hz, 1H), 8.41 (d, *J* = 1.8Hz, 1H), 8.24 (s, 1H), 8.21 (s, 1H), 7.96 (dd, *J* = 2.2, 8.4 Hz, 1H), 7.86 (d, *J* = 8.4 Hz, 1H), 7.76 (d, *J* = 9.4 Hz, 1H), 7.57 (dd, *J* = 1.8 Hz, 9.4 Hz, 1H), 7.23 (t, *J* = 4.9 Hz, 9.4Hz, 1H), 5.45 (s, 2H), 4.46 (q, *J* =7.1, 2H), 1.44 (t, *J* = 7.1 Hz, 3H).

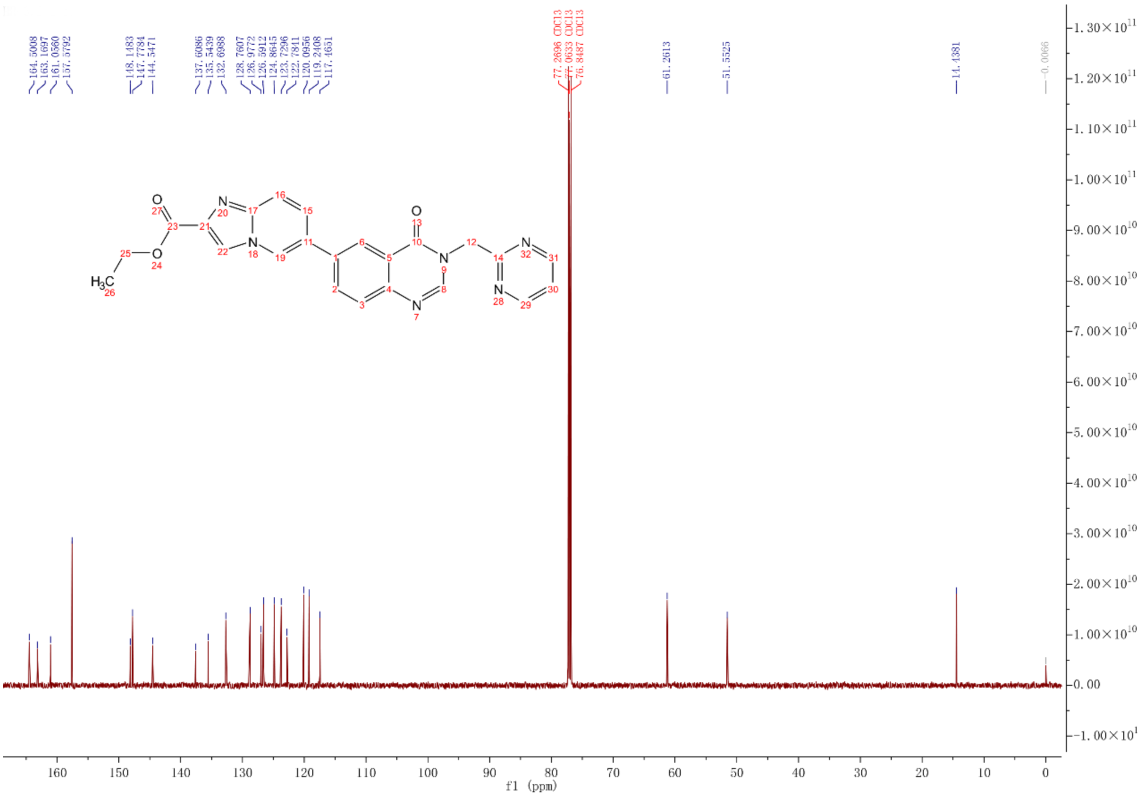


**Figure S2.** $^{13}$**C-NMR spectra**

$^{13}$C-NMR (151 MHz, $CDCl_3$) δ 164.50, 163.17, 161.06, 157.58, 148.15, 147.78, 144.54, 137.61, 135.54, 132.70, 128.76, 126.98, 126.59, 125.0, 123.8, 122.9, 120.2, 119.3, 117.6, 61.4, 51.7, 14.5.

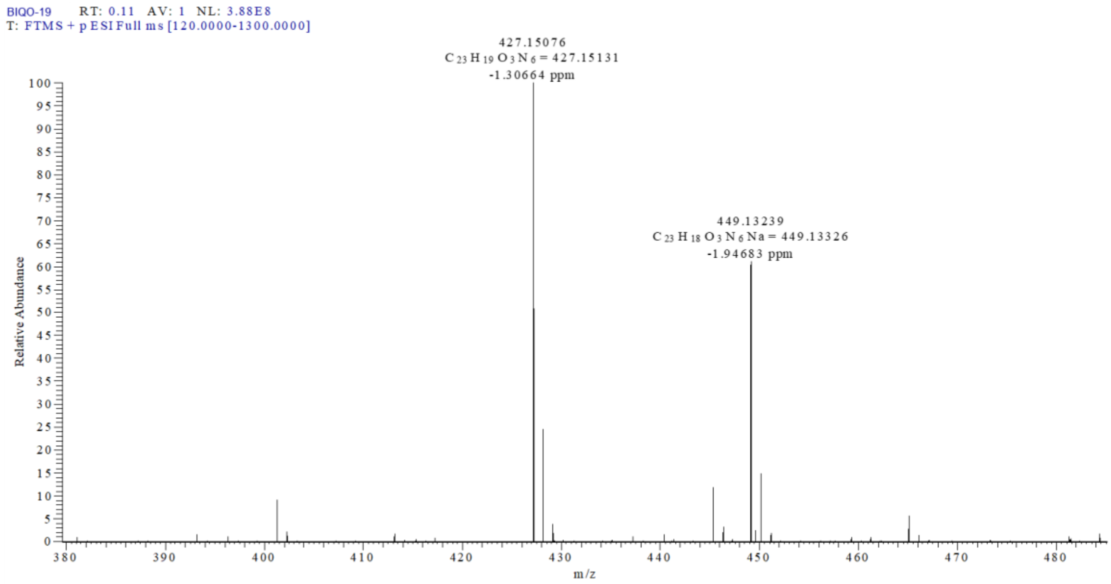


**Figure S3. High resolution mass spectra**

HRMS (ESI-QTOF) m/z Calcd. For $C_{23}H_{19}O_3N_6$ $[M+Na]^+$ m/z: 449.1333, found: 449.1324.
